# Case report: Type IIIa endoleak due to disconnection of AFX2 and the Endurant aortic extension cuff after endovascular aneurysm repair

**DOI:** 10.1016/j.ijscr.2025.111625

**Published:** 2025-07-07

**Authors:** Rina Ebe, Yoshikatsu Nomura, Ryota Kawasaki, Yutaka Koide, Hiroshi Tanaka, Hirohisa Murakami

**Affiliations:** a Department of Cardiovascular Surgery, Hyogo Prefectural Harima-Himeji General Medical Center, 3-264 Kamiya-cho, Himeji, Hyogo 670-8560, Japan; b Department of Radiology, Hyogo Prefectural Harima-Himeji General Medical Center, 3-264 Kamiya-cho, Himeji, Hyogo 670-8560, Japan

**Keywords:** Type IIIa endoleak, Disconnection, AFX2

## Abstract

**Introduction:**

Type IIIa endoleak (TIIIaEL) is a serious complication following endovascular aneurysm repair, requiring immediate reintervention. We describe a case of TIIIaEL with proximal component separation in a patient treated with an AFX2 endograft.

**Presentation of case:**

TIIIaEL and an enlarged aneurysm were observed in an 87-year-old man 30 months after endovascular repair of an abdominal aortic aneurysm using an Endurant aortic extension cuff and an AFX2. The initial aneurysm diameter was 58 mm. Following surgery, the patient was discharged without complications. Follow-up was conducted with computed tomography every 6 months. The aneurysm decreased by 1–2 mm at the 24-month postoperative visit but subsequently showed gradual disconnection of the junction, leading to a diagnosis of aneurysm enlargement. After TIIIaEL, the aneurysm diameter was 62 mm. Urgent reintervention was performed through endovascular treatment instead of open surgery. Two non-bare VELA Proximal Endografts (A34–34/C100V, Endologix, Irvine, CA, USA) and one Excluder aortic extender (PLA280300J, W. L. Gore & Associates Inc., Flagstaff, Arizona, USA) were inserted into the gap between the proximal cuff and main body. No endoleaks were observed. The aneurysm diameter decreased without complications. No recurrence was documented after 1 year. The patient is progressing without noticeable symptoms.

**Discussion:**

The AFX-aortic extension cuff junction may become disconnected due to self-expanding movement and linear forces, causing TIIIaEL. Devices from different manufacturers may have contributed to the occurrence of TIIIaEL.

**Conclusion:**

Modular device overlaps should be as long as possible, and close postoperative monitoring is necessary when this device is used.

## Highlights

• Type IIIa endoleak (TIIIaEL) is a serious post-aneurysm repair complication.
• An 87-year-old man had TIIIaEL with proximal component separation after AFX2 use.
• Urgent endovascular reintervention was chosen over open surgery to seal the leak.
• Using devices from different manufacturers may lead to TIIIaEL.
• Sufficient device overlapping and close monitoring are vital to prevent TIIIaEL.

## 1 Introduction

The AFX2 (Endologix, Irvine, CA, USA) is a unique bifurcated unibody endograft specifically for narrow aortic bifurcations and the only abdominal stent graft device that can be placed in the endoskeleton. Its ActiveSeal™ mechanism enables graft adherence to the aneurysm wall, potentially preventing type II endoleaks. Conventional stent-grafts typically anchor at the proximal portion of the aneurysm, whereas the AFX device employs a distal fixation at the iliac arteries, making it advantageous in cases with a short proximal neck [1]. Some features of the device can cause type IIIa endoleak (TIIIaEL) from loss of junction during stenting. Type III endoleak is a unique complication of endovascular aneurysm repair (EVAR), with an estimated incidence of 3.0 %–4.5 % [2]. In TIIIaEL, separation of graft components or inadequate apposition leads to pressurization of the aneurysm sac, prompting the U.S. Food and Drug Administration (FDA) to issue a warning in December 2022. TIIIaEL requires immediate reintervention upon diagnosis due to the increased risk of rupture from high-pressure endoleaks [3]. Herein, we report a case of junction disconnection between an AFX2 and an aortic extension cuff from a different manufacturer 30 months postoperatively, resulting in a TIIIaEL. Endovascular treatment was performed with a favorable outcome. This case report has been reported in line with the SCARE checklist [4].

## 2 Presentation of case

**Fig. 1:**
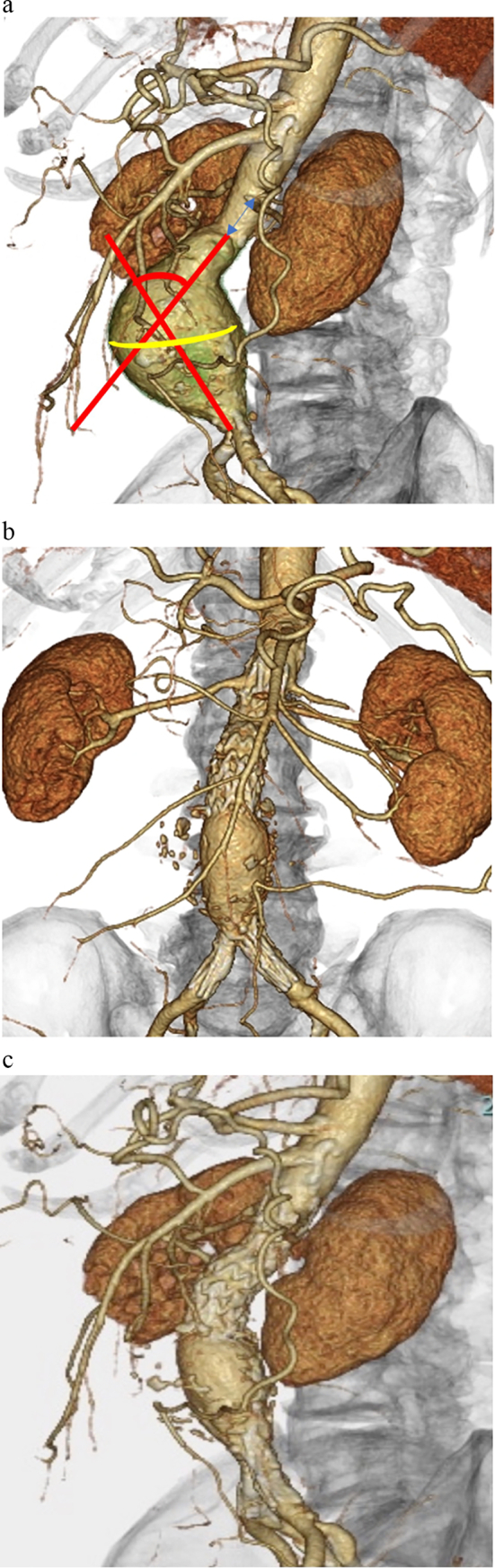
(A) Preoperative computed tomography (CT) shows an abdominal aortic aneurysm measuring 58 mm in diameter, with a proximal neck 20 mm in diameter and 15.6 mm in length. The proximal neck angulation is 60° in the anteroposterior direction. (B) (C) Postoperative CT shows a 25-mm overlap between the Endurant aortic extension cuff and main body.

**Fig. 2:**
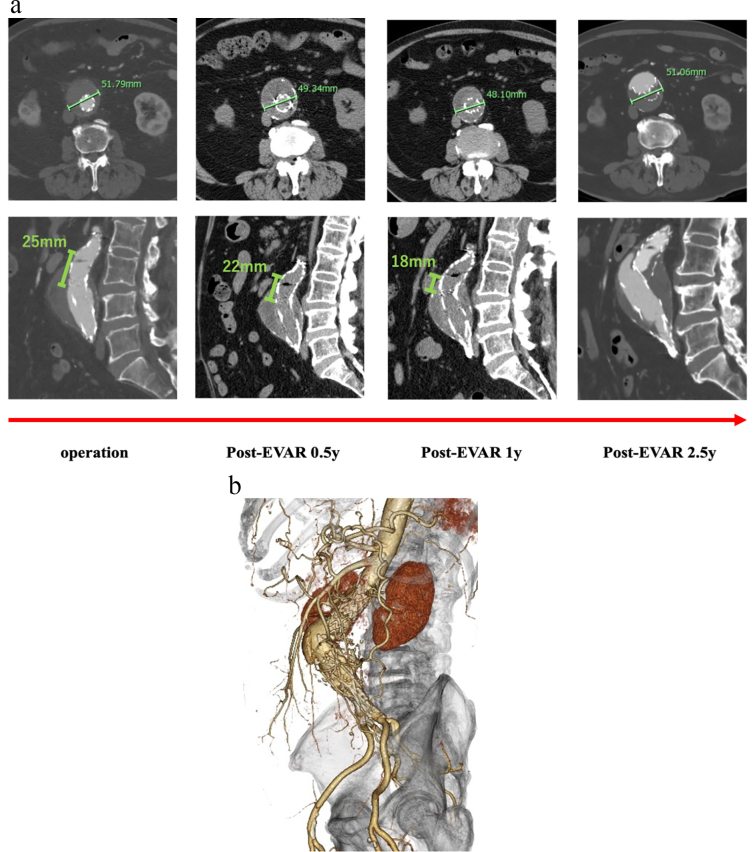
(A) (B) Postoperative serial computed tomography images reveal a 1–2 mm decrease in aneurysm diameter and disconnection caused by progressive loss of component overlap.

An 87-year-old man who underwent EVAR for an abdominal aortic aneurysm (AAA) 30 months ago presented with AAA expansion observed on computed tomography (CT) angiography. Preoperatively, the AAA was 58 mm in diameter, with a proximal neck 20 mm in diameter, 16 mm in length, and angulated 60° in the anteroposterior direction (Fig. 1A). EVAR was performed using an Endurant extension cuff (ETTF2323C70EJ; Medtronic, Minneapolis, MN, USA) and AFX2 (BEA 28–90/I16–30) (Fig. 1B). The Endurant was chosen for easier positioning, considering the short proximal neck from the aortic bifurcation to the renal arteries. The AFX2 was selected to reduce the risk of a type II endoleak through the ActiveSeal™ mechanism. Moreover, the narrow aortic bifurcation in this case was considered well suited for the AFX2. To prevent TIIIaEL, an Endurant cuff was first placed below the renal artery, followed by a size-up AFX2. At our institution, placement of exoskeletal devices is prioritized when devices from different manufacturers are used, because exoskeletal devices do not expand, whereas endoskeletal devices expand to achieve proper sealing, potentially reducing TIIIaEL occurrence. Balloon dilatation was performed at the proximal and distal landings and the junction. Final angiography and postoperative enhanced CT revealed no endoleaks. Postoperative CT showed a 25-mm overlap between the aortic extension cuff and main body. Postoperative CT performed every 6 months showed a 1–2-mm decrease in aneurysm diameter at 1, 6, 12, and 24 months. However, a later review showed gradual junction disconnection, leading to diagnoses of aneurysm enlargement and TIIIaEL at 30 months postoperatively (Fig. 2). The patient was in good condition and clinically stable, with no relevant symptoms. Although the junction was not completely disconnected, a thrombus in the Endurant extension impaired blood flow to the lower extremities. Ankle-brachial pressure indices of both lower extremities, previously above 1.0, decreased to 0.6. The aneurysm diameter also increased from 59 to 62 mm, necessitating immediate reintervention. Endovascular treatment was chosen over open surgery due to the patient's advanced age and frailty.

Three days after CT scanning, reintervention was performed under general anesthesia. Because a large thrombus within the proximal cuff raised concerns about embolization, the bilateral common femoral arteries were clamped to occlude distal flow during EVAR and prevent embolism. Due to difficulty passing the wire retrograde through the gap between the Endurant cuff and the AFX2, a transbrachial approach was selected. A 60-cm guiding sheath was inserted via the left brachial artery, and a guidewire was inserted anterogradely into the AFX2 main body. A 4Fr sheath was inserted into the left brachial artery and subsequently replaced with a 60-cm Parent Plus 45 Catheter (Medikit Co., Ltd., Tokyo, Japan). The descending aorta was selected using an RC09 catheter. Subsequently, a Bernstein catheter was introduced and advanced through the gap between the Endurant extension and AFX2 main body without difficulty.

**Fig. 3:**
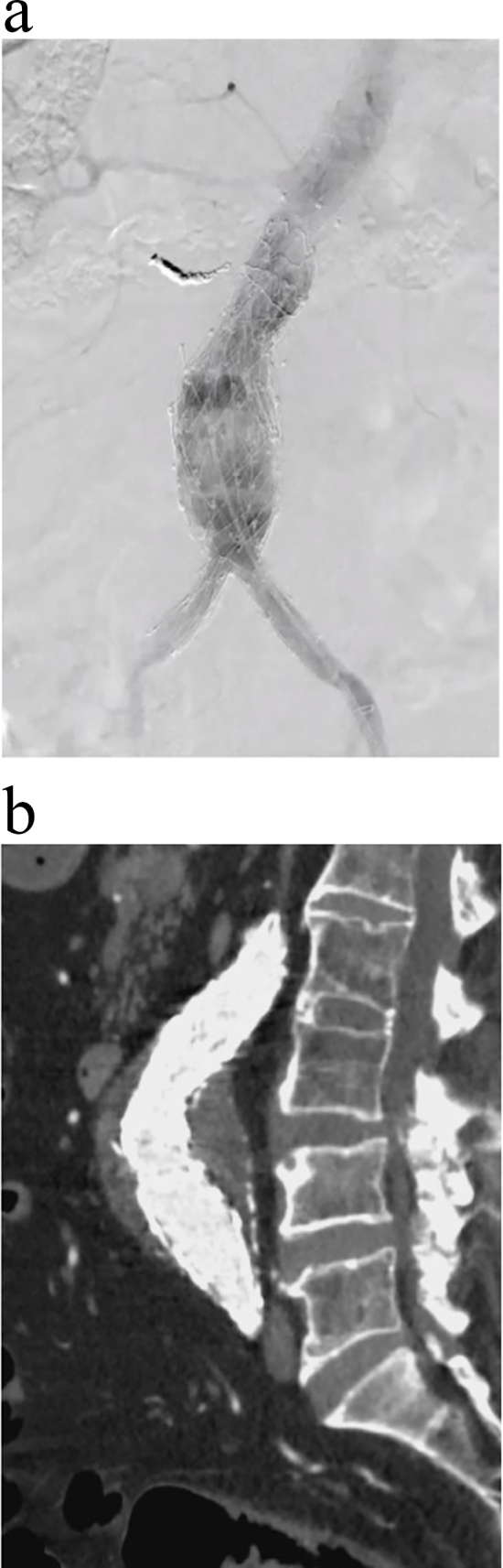
(A) No endoleaks are observed on completion of angiography. (B) Postoperative computed tomography (CT) scan.

An 8Fr sheath was placed in the left common femoral artery. A 300-cm, 0.9-cm diameter Radifocus wire (Terumo, Tokyo, Japan) was inserted through the brachial artery using the Indy OTW™ Vascular Retriever (Cook Medical, Bloomington, IN, USA), as retrograde passage through the gap was considered too difficult. The 8Fr sheath was replaced with an AFX sheath. During the initial EVAR, an Endurant extension was used, resulting in the elongation of the proximal neck. Therefore, for the additional EVAR, VELA Proximal Endografts were selected to ensure consistency with the manufacturer. Initially, a non-bare VELA Proximal Endograft (A34–34/C100V, Endologix) was implanted from the terminal aorta to the Endurant proximal extension, followed by balloon touch-up. However, the junction length was insufficient, requiring implantation of an additional VELA Proximal Endograft proximal to the Endurant extension. As proximal junction infolding was possible, the sheath was replaced, and an Excluder aortic extender (PLA280300J, W. L. Gore & Associates Inc., Flagstaff, Arizona, USA) was implanted. The endoskeleton structure of the AFX2 and VELA endografts resulted in connections only at the edges, increasing the likelihood of wrinkling. Therefore, an Excluder cuff was used to secure the graft. No endoleaks were observed upon completion of angiography (Fig. 3A–B).

The postoperative course was uneventful, and the patient was discharged on postoperative day 10. At the 1-year follow-up, no recurrence of TIIIaEL was observed, and the aneurysm had decreased in size.

### 2.1 Imaging modalities

Preoperative diagnosis using CT revealed an infrarenal AAA and enabled evaluation of the aortic neck and iliac artery anatomy. Intraoperative imaging used fluoroscopic guidance with digital subtraction angiography to ensure accurate device positioning and confirm aneurysm exclusion. Postoperative follow-up included contrast-enhanced CT at 1 week, 6 months, and 1 year to assess for endoleak, graft integrity, and aneurysm size. No endoleak was observed early postoperatively, and sac regression was confirmed during follow-up.

## 3 Discussion

The ActiveSeal™ mechanism is a distinctive feature of the AFX2 that allows stent-graft adhesion to the vessel wall via differential pressure between the graft interior and the aneurysm [1]. When using the AFX2, the curvature angle of the aneurysm directly correlates with linear forces acting to separate the central cuff from the main body. Patients with larger curvature angles are more prone to this effect, making adherence to usage guidelines and long-term follow-up essential. Therefore, sufficient overlap between the aortic cuff and main body is necessary when using AFX, and the manufacturer recommends exceeding the aneurysm length by 20 mm when upgrading to AFX2 [5]. Although TIIIaEL occurrence with AFX has rarely been reported [2,6], mid-term occurrence is a concern with AFX2. While the unibody design of AFX2 provides flexibility and expandability, it lacks strength and deforms easily. Additionally, stent-grafts do not fully adhere to the vessel wall if an AAA is present, increasing the risk of migration and TIIIaELs compared to other models. In larger aortic aneurysms, the ActiveSeal™ mechanism expands the main body distally, increasing linear force between the main body and aortic cuff, potentially causing TIIIaEL. In 2020, 2021, and 2022, the FDA issued recommendations regarding TIIIaEL risk with AFX2, suggesting alternative devices be used when possible [3]. If AFX2 is selected, annual follow-up is recommended. Additionally, the efficacy and safety of combining AFX2 with devices from other manufacturers have not been evaluated.

In this case, a 25-mm overlap was inadequate for a maximum aneurysm radius of 58 mm; the Instruction for Use (IFU) indicates a minimum overlap of 50 mm. The proximal neck angle was 60°, the upper limit specified in the IFU. The AFX2 tends to straighten, increasing the risk of TIIIaEL in patients with angulated proximal necks. Although the FDA has not evaluated combining an Endurant extension cuff with AFX2 or devices from different manufacturers, such use was unavoidable in this case. Successful treatment with favorable outcomes using a combination of AFX2 and Endurant cuff has been reported in cases with narrow aortic bifurcation and challenging proximal neck [7]. Although combining devices from different manufacturers may be a valuable therapeutic option for complex cases, it may also increase the risk of TIIIaEL. The overlap required by the IFU, corresponding to the maximum aneurysm radius plus 20 mm, was not achieved. Additionally, the angle reached the upper limit specified by the IFU, possibly contributing to component separation. Extending the overlapping zone could potentially prevent TIIIaEL. Moreover, we had not previously encountered TIIIaEL associated with AFX use; long-term follow-up of AFX2-treated cases is crucial.

## 4 Conclusion

In conclusion, we observed a proximal TIIIaEL in a patient following AFX2 use. Proximal cuff disconnection may have resulted from combining the AFX2 with a device from a different manufacturer and insufficient overlap, leading to TIIIaEL. FDA recommendations and limited reports on TIIIaEL highlight the importance of proper device use. Avoiding the use of devices from different manufacturers and ensuring sufficient overlap are crucial to preventing severe complications. Studies with early to midterm follow-up are required to further evaluate the long-term safety and performance.

## Ethical approval and informed consent

This case was managed in accordance with the ethical standards of the Hyogo Prefectural Harima-Himeji General Medical Center and the 1964 Declaration of Helsinki and its subsequent amendments. Institutional ethics committee approval was not required for this type of case report, as per institutional policy. Written informed consent was obtained from the patient for publication of this case report and accompanying images. Patient confidentiality and anonymity have been strictly maintained, and no identifying information is included in the manuscript. A copy of the written consent is available for review by the Editor-in-Chief upon request.

## Guarantor

Yoshikatsu Nomura.

## Author contributions

Conception and Design: RE, YN; Data collection: RE, YN, RK; Writing: RE; Critical review and revision: all authors; Final approval of the article: all authors; Accountability for all aspects of the work: all authors.

## Declaration of Generative AI and AI-assisted technologies in the writing process

Generative AI and AI-assisted technologies were NOT used in the preparation of this work.

## Sources of funding

This research received no specific grant from any funding agency in the public, commercial, or not-for-profit sectors.

## Declaration of competing interest

The authors declare no conflicts of interest.

## Data Availability

In accordance with the IJS Case Reports data-sharing policy, the datasets supporting the findings of this study are available in a public repository. This case report has been reported in line with the SCARE checklist.

## Appendix A Supplementary data

Supplementary video This video demonstrates the surgical procedure. A Radifocus wire was inserted through the left brachial artery, followed by a snare catheter inserted through the left common femoral artery for the pull-through technique. Aortic angiography confirmed separation between the Endurant extension and AFX2 main body, with a type IIIa endoleak (TIIIaEL) present. The sheath in the left common femoral artery was exchanged, and two non-bare VELA Proximal Endografts along with one Excluder aortic extender were placed, completing the procedure. Supplementary video
